# The prevalence and root causes of surgical site infections in public versus private hospitals in Ethiopia: a retrospective observational cohort study

**DOI:** 10.1186/s13037-019-0206-4

**Published:** 2019-07-10

**Authors:** Kidanie Fisha, Muluken Azage, Getasew Mulat, Koku Sisay Tamirat

**Affiliations:** 1 Feleghiwote Comprehensive Specialized Hospital, Bahir Dar, Ethiopia; 2 School of Public Health College of Medicine and Health Sciences, Bahir Dar University, Bahir Dar, Ethiopia; 3 Department of public health, GAMBY Medical and Business College, Bahir Dar, Ethiopia; 4 Department of Epidemiology and Biostatistics Institute of Public Health, College of Medicine and Health Science, University of Gondar, Gondar, Ethiopia

**Keywords:** Surgical site infection, Root causes, Private versus public hospital, Ethiopia

## Abstract

**Background:**

Healthcare-associated illnesses, of which surgical site infection is the most common are significant causes of morbidity and mortality. Therefore, this study aimed to determine the prevalence and root causes of surgical site infections in public versus private hospitals in Ethiopia.

**Methods:**

An institution based retrospective observational cohort study was conducted among patients who underwent surgical procedures at public and private health facilities from March 15 to April 15, 2018. Samples were selected by the simple random sampling technique, and data extracted from the patient’s medical chart, operation, and anesthesia notes. Data were entered using Epi info version 7 and analyzed using STATA 14. Binary logistic regression was fitted to identify factors associated with surgical site infections in private and public hospitals. Crude and adjusted odds ratios (OR) with a 95% confidence interval (CI) were computed to assess the strength of associations. Variables with a *p*-value less than 0.05 in the multivariable logistic regression model considered as significant predictors of surgical site infections.

**Result:**

The overall prevalence of surgical site infections was 9.9% (95%CI: 7.8, 12.5). The prevalence of the infections was higher in procedures performed in public hospitals (13.4%) compared to private hospitals (6.5%). Rural residence (AOR = 0.13, 95%CI: 0.034 0.55), clean-contaminated and dirty wound (AOR = 12.81, 95%CI: 4.42 37.08) were significant predictors of the infections in private hospitals. Similarly, clean-contaminated and dirty wounds (AOR = 4.37, 95%CI: 1.88 10.14), length of hospital stay≥6 days (AOR = 2.86, 95%CI: 1.11 7.33), and surgical operation time of over 1 h (AOR = 15.24, 95%CI: 4.48 51.83) were such factors in public hospitals.

**Conclusion:**

The prevalence of surgical site infections was high, and significant differences were also observed between public and private hospitals. Clean-contaminated and dirty wounds, prolonged operation, and length of hospital stay were predictors of surgical site infections among patients in public hospitals, whereas clean-contaminated wound and rural dwellings were predicted the infections among patients operated in the private hospital.

## Background

Healthcare-associated infections (HIAs) are a significant source of preventable morbidity and mortality [1–3]. More than 30% of the HIA are surgical site infections (SSI) defined as infections related to operative procedures that occur at or near surgical incisions within 30 days of the procedure or within 90 days if prosthetic materials are implanted at surgery [4]. Surgical site infections may extend from the skin and superficial subcutaneous tissues of incision sites to deep subcutaneous tissues and organ spaces [5]. Due to poor infection prevention practices among health care facilities in low and middle-income countries, the incidence of SSI is substantially higher than in high-income countries [6, 7].

SSI posed a significant clinical and financial burden on patients. Those who developed post operation SSI had 2–11 times higher risk of mortality [8]. Moreover, SSIs had a wide range of effects on patients and health care systems like; patient discomfort, prolonged hospital stay, and more days off work. In addition, SSI increased the cost of therapy, and the cost of an operation catastrophically raise by 300 to 400% [9, 10].

Surgical site infections are attributable to a variety of factors which can be classified into patient-related, procedure-related and others. Other risk factors include the volume of surgeries performed in the department, the season, the working environment in the operation room, and the indications for surgery [3, 6, 8, 11]. The World Health Organization (WHO) and other studies indicated that periodic surveillance and feedback for surgeons on SSIs rate and associated factors can decrease up to 50% of cases [12].

Government stakeholders such as the Federal Ministry of Health of Ethiopia (FMOH) and different health sector stakeholders incorporated surgical site infections with hospital key performance indicators (KPI) for continued surveillance of the conditions. Also, infection prevention and patient safety training and updates need to be continuously given to health care workers. Though some studies/clinical audits have been conducted in other sub-Saharan countries, there has been scarce evidence that compares the prevalence of surgical site infections between public and private hospitals in Ethiopia.

Therefore, our study aimed to determine the prevalence and root causes of surgical site infections in public versus private hospitals of Ethiopia. This study could provide insight into surgical site infections to different organizations and could help in evidence-based interventions in health facilities.

## Methods

### Study design

An institution based retrospective observational cohort study was carried out in public and private hospitals of Bahir Dar city, Ethiopia, from March 15 to April 15, 2018. Bahir Dar is the capital city of the Amhara National Regional State, located 565 km northwest of Addis Ababa, Ethiopia. The city has two public and two private hospitals which serve the people in the city and nearby districts. Of the hospitals, Feleghiwot comprehensive specialized is public, and ADINAS and GAMBY are private hospitals included in the study. The public and private hospitals had a total of 436 and 69 beds, respectively with general surgical and orthopedics, gynecological and obstetric operation rooms. Twenty surgeons, 63 residents and general practitioners (GPs), 12 anesthetists, and 69 nurses worked in the surgical departments of the public and private hospitals. On average, more than 8,000 patients got admitted into the surgical, gynecology and orthopedic wards of the public hospitals each year. Similarly, more than 5,000 patients got surgical services in private hospitals annually.

### Patient population

All patients admitted and underwent a surgical procedure in the public and private hospitals of the city were the source population. Patients who underwent elective and emergency major operations and admitted into the surgical, orthopedics, gynecology and obstetrics wards of the hospital during the study were included in the study. Patients who underwent re-operations and had contaminated and dirty operations, neonates and included patients who showed signs and symptoms of infection within 48 h of admission and patients admitted for less than 48 h were excluded from the study.

The sample size was determined using two population proportions with the assumptions of surgical site infection of 50% in the public and 38% in the private hospitals and 80% power with a 1:1 ratio. The final calculated sample size was 642 study participants (321 patients from each type). Initially, one public and two private hospitals were selected. Of the two public hospitals in the city only one of the public hospital that was Feleghiwote Comprehensive specialized public hospital selected with its 321 patients. This hospital is the referral site for the surgical procedure from lower health facilities and primary hospitals. Furthermore, Samples were proportionally assigned to the private hospitals accordingly to the number of patients operated on there. Thus, 158 and 163 patients were selected from ADINAS and GAMBY teaching hospitals, respectively. The final samples/patients were selected using the simple random sampling technique from hospital registration databases and operation logbooks.

### Data collection and analysis

A pre-tested and structured questionnaire was used to collect data adopted from a variety of literature. Patient socio-demographic characteristics, clinical profiles and surgical procedure relating characteristics were collected from patient medical records and surgeons operation and anesthesia notes. Three clinical nurses and one supervisor were involved in the data collection process. The training was given for 1 day on how to select study participants and review medical documents. Before the actual data collection, the questionnaire was pre-tested in another hospital, appropriate corrections were made according to the findings. The research team supervised the data collection process daily.

Surgical site infection following surgical procedures was the outcome variable in both public and private hospitals. Socio-demographic variables (age, sex, residence, and average monthly income), clinical characteristics (wound type, ASA score, length of hospital stay, duration of surgical operation and the surgeon, co-morbidities) were independent variables. Surgical site infection: An infection related to an operative procedure that occurs at or near the surgical incision within 30 days of the procedure or within 90 days if prosthetic material is implanted at the surgery.

The collected data were edited, coded and entered into Epi-info version 7 and export to SPSS version 20 software for further data management and analysis. Summary measures like percentage, means, and standard deviation were computed and the summarized data were presented in tables, graphs, and charts for both the private and public hospitals. A binary logistic regression model was fitted for overall and independently for the public and private hospitals to identify factors associated with surgical site infection. Crude and adjusted odds ratios (OR) with a 95% CI were computed to assess the strength of association between the independent and outcome variable. Variables with a *p*-value of less than 0.05 were considered as statistically significant predictors of surgical site infections.

## Results

### Socio-demographic characteristics

A total of 642 patients who underwent surgical procedures were included (321 from each hospital). The median age of patients was 33 (IQR: 25–46) years; about 43.9% of them were aged ≥36 years. More than half (54.2%) of the patients in the public, 60.9% in private hospitals were female. Similarly, 54.5% in the public hospital and 70.1%% in the private hospital were rural and urban dwellers, respectively (Table 1).

**Table 1:** socio-demographic characteristics of clients among public and private hospital Bahir Dar Amhara Region, June 2018 (*n* = 642)

Variable	Public hospital no (%) *n* = 321	Private hospital no (%) *n* = 321	Total no (%) *n* = 642
Age in years			
< =24	70(21.8%)	76(23.67%)	146(22.7%)
25–35	128(39.87%)	86(26.79%)	214(33.4%)
> =36	123(38.3%)	159(49.5%)	282(43.9%)
Monthly income (ETB)			
< =1600	111(34.6%)	60(18.7%)	171(26.6%)
1601–2500	88(27.4%)	79(24.6%)	167(26.01%)
> =2501	122(38.0%)	182(56.7%)	304(47.35%)
Sex			
Male	126(39.3%)	147(45.8%)	273(42.5%)
Female	195(60.7%)	174(54.2%)	369(57.5%)
Residence			
Urban	146(45.5%)	225(70.1%)	371(57.78%)
Rural	175(54.5%)	96(29.9%)	271(42.2%)

Elective surgery was the most common (64.5%) procedure in the private and 59.85% in the public hospitals. Regarding chronic comorbidities 13.4% of participants had one or more co-morbidities of which DM, hypertension and HIV were the most common. Clean wound type was the most common (76.3%) the in public and (88.2%) in the private hospitals. Abdominal laparotomy of which 33.3 and 55.7% were performed in the public and private hospitals, respectively was the most common procedure. The majority of the surgical procedures were done by surgeons in the private hospitals and residents and general practitioners in the public hospital, 98.4 and 71.1%, respectively. Regarding the duration of surgical procedures, 58.88% operation in the public and 41.43% in the private hospitals lasted 30–60 min (Table 2).

**Table 2:** Clinical characteristics of patients undergone a surgical procedure in Bahir Dar city public and private hospitals, Ethiopia, June 2018 (*n* = 642)

Variable	Public Hospital (*N* = 321)	Private Hospital (*N* = 321)	Total (*N* = 642)
Type of surgery			
Elective	192 (59.8%)	207 (64.5%)	399 (62.15%)
Emergency	129 (40.2%)	114 (35.5%)	243 (37.85%)
Given antibiotic prophylaxis			
Yes	305 (95.0%)	306 (95.3%)	611 (95.2%)
No	16 (5.0%)	15 (4.7%)	31 (4.8%)
Timing of prophylactic drug administration			
Before surgery	305 (95.0%)	275 (85.7%)	580 (90.39%)
After surgery	16 (5.0%)	46 (14.3%)	62 (9.65%)
ASA score			
Class1	162 (50.5%)	301 (93.8%)	463 (72.1%)
Class2	132 (41.1%)	17 (5.3%)	149 (23.2%)
Class3	22 (6.9%)	3 (0.9%)	25 (3.9%)
Class4	5 (1.6%)	0	5 (0.8%)
Substance use			
No	305 (95.0%)	296 (92.2%)	601 (93.6%)
Yes	16 (5.0%)	25 (7.8%)	41 (6.4%)
Types of substance use			
Alcoholic	4 (1.2%)	15 (4.7%	19 (2.95%)
Smoker	12 (3.8%)	10 (3.1)	223.4%)
Had co-morbidities			
No	270 (84.1%)	286 (89.1%)	556 (86.6%)
Yes	51 (15.9%)	35 (10.9%)	86 (13.39%)
Type of co-morbidities			
Diabetes mellitus	10 (3.1%)	14 (4.4%)	24 (3.7%)
HIV	8 (2.5%0	6 (1.9%)	14 (2.1%)
Hypertension	1 (4.7%)	11 (3.4%)	12 (1.9%)
Malignancy	13 (4.0%)	9 (2.850	22 (3.4%)
Antiseptic used for skin preparation			
Iodine	31 (9.7%)	20 (6.2%)	51 (7.9%)
Isopropyl alcohol l plus iodine	237 (73.8%)	266 (82.9%	503 (78.34%)
Isopropyl alcohol	53 (16.5%)	35 (10.9%)	88 (13.7%)
provider doing the procedure			
Surgeon	93	316	409 (63.7%)
Resident and GP	228	5	233 (36.3%)
Type of surgery			
Neuro surgery	33 (10.3%)	32 (10.0%)	65 (10.12%)
Abdominal Surgery	114 (35.5%)	179 (55.8%)	293 (45.6%)
Chest surgery	23 (7.2%)	6 (1.9%)	29 (4.5%)
Genitourinary Surgery	23 (7.2%)	31 (9.75)	54 (8.4%)
Cesarean section Surgery	128 (39.9%)	73 (22.7%)	201 (31.3%)
Type of dressing			
Gauze	179 (55.8%)	33 (10.3%)	212 (33.02%)
Gauze and saline	79 (24.6%)	264 (82.2%)	343 (53.4%)
Gauze and Iodine	63 (19.6%)	24 (7.5%)	87 (13.6%)
Duration of operation			
within 30 min	75 (23.36%)	140 (43.6%)	215 (33.48%)
31-60 min	189 (58.87%)	133 (41.43%)	322 (50.15%)
≥ 60 min	57 (17.76%)	48 (14.95%)	105 (16.36)
Wound type			
Clean	245 (76.32%)	242 (75.38%)	487 (82.2%)
Contaminated and dirty	76 (23.675)	79 (24.61%)	155 (3.4%)
Length of stay at hospital			
< 6 days	144 (44.86%)	190 (59.19%	334 (52.02)
≥ 6 days	177 (55.14)	131 (40.80%)	308 (47.98)
Got blood transfusion			
Yes	26 (8.10%)	80 (24.92%)	106 (16.51%)
No	295 (91.90%)	241 (75.08%)	536 (83.49%)

### Prevalence of surgical site infections

The overall prevalence of surgical site infections at both public and private hospitals was 9.9% with (95%CI: 7.8 to 12.5%). The prevalence of surgical site infection in the public hospital was 13.4% (95%CI: 9.7–17.1%) and 6.5% (95%CI: 4.0–9.3%) in private hospitals.

### Factors associated with surgical site infection following procedures

We fitted three different models to identify factors associated with surgical site infections in both and public versus private hospitals.

The first model was fitted to assess the overall factors of surgical site infection in all hospitals. Variables such as wound type, average monthly income, length of hospital stay, a physician who performed the procedure, and duration of procedure were significantly associated with surgical site infections in hospitals. Accordingly, for patients who had contaminated and dirty wounds, the odds of surgical site infections were 5.35 times higher compared to those who had clean wounds [AOR = 5.35, 95%CI (2.84 10.06)]. Similarly, average monthly income of [1601–2500 ETB (AOR = 2.88, 95%CI: 1.15 7.18) or ≥ 2501 ETB (AOR = 2.54, 95%CI: 1.05 5.99)] and length of hospital stay≥6 days (AOR = 1.96, 95%CI: 1.03 3.71) were more likely to result in surgical site infections compared to their counterparts. Furthermore, surgical operation duration of above 1 h (AOR = 5.36, 95%CI: 2.09 13.7) and procedures are done by residents and general practitioners (AOR = 3.99, 95%CI: 2.01 7.91) were more likely to cause surgical site infections compared to their counterparts (Table 3).

**Table 3:** Binary logistic regression analysis to identify factors associated with surgical site infection from overall hospitals in Bahir Dar city, Ethiopia, May 2018(*n* = 642)

Characteristics	Surgical site infection		COR(95%CI)	AOR(95% CI)
	Yes	No		
Age of patient				
≤ 24	7	139	1	1
25–35	14	200	1.39 (0.54 3.53)	1.35 (0.48 3.77)
≥ 36	43	239	3.57 (1.56 8.15)	2.32 (0.90 6.59)
Sex of patients				
Male	36	237	1	1
Female	28	341	0.54 (0.32 0.91)	0.59 (0.32 1.09)
Wound type				
Clean	27	460	1	1
Clean-contaminated and dirty	37	118	5.34 (3.12 9.12)	5.35 (2.84 10.06)*
Monthly income in ETB				
≤ 1600	10	161	1	1
1601–2500	22	145	2.44 (1.11 5.33)	2.88 (1.15 7.18)*
≥ 2501	32	272	1.89 (0.90 3.95)	2.54 (1.05 5.99)*
Length of hospital stay				
< 6 days	18	316	1	1
≥ 6 days	46	262	3.08 (1.74 5.44)	1.96 (1.03 3.71)*
Duration of surgical procedure				
≤ 30 min	9	206	1	1
31–60 min	28	294	2.17 (1.007 4.71)	1.21 (0.49 2.99)
> 60 min	27	78	7.92 (3.56 17.60)	5.36 (2.09 13.75)*
Procedure done by				
Surgeon	25	384	1	1
Residents and GP	39	194	3.08 (1.81 5.25)	3.99 (2.01 7.91)*
Co-morbidities				
No	49	507	1	1
Yes	15	71	2.18 (1.16 4.10)	1.31 (0.62 2.77)

*Shows *P*-value less than 0.05

The second model was fitted for patients who underwent a surgical procedure in public hospitals only. Accordingly, wound type, length of hospital stay and duration of operation were factors associated with surgical site infections in public hospitals at a *p*-value of 0.05. Patients who had clean-contaminated and dirty wounds [AOR = 4.37, 95%CI (1.88 10.14), length of hospital stay≥6 days (AOR = 2.86, 95%CI: 1.11 7.33), and surgical operation lasting above 1 h (AOR = 15.24, 95%CI: 4.48 51.83) were more likely to end in surgical site infections compared to their counterparts (Table 4).

**Table 4:** Binary logistic regression analysis to identify factors associated with surgical site infection among patients who undergone surgical procedure in public hospital of Bahir Dar city, Ethiopia, May, 2018(*n* = 321)

Characteristics	SSI		COR(95%CI)	AOR(95% CI)
	Yes	No		
Age in years				
≤ 24	4	66	1	1
25–35	9	119	1.24 (0.37 4.20)	1.33 (0.35 5.00)
≥ 36	30	93	5.32 (1.78 15.82)	3.28 (0.98 10.99)
Sex				
Male	23	103	1	1
Female	20	175	0.51 (0.26 0.97)	0.86 (0.36 1.89)
Wound type				
Clean	20	225	1	1
Clean contaminated and dirty	23	53	4.88 (2.49 9.53)	4.37 (1.88 10.14)*
Monthly income in ETB				
≤ 1600	9	102	1	1
1601–2500	15	73	2.32 (0.96 5.61)	2.42 (0.84 6.90)
≥ 2501	19	103	2.09 (0.90 4.83)	2.18 (0.82 5.76)
Length of hospital stay				
< 6 days	7	137	1	1
≥ 6 days	36	141	4.99 (2.15 11.61)	2.86 (1.11 7.33)*
Duration of surgical procedure				
≤ 30 min	5	70	1	1
31–60 min	17	172	1.38 (0.49 3.89)	2.52 (0.82 7.79)
> 60 min	21	36	8.16 (2.84 23.45)	15.24 (4.48 51.83)*

*Shows *P*-value less than 0.05

The third model was fitted for patients who underwent a surgical procedure in private hospitals only. Variables such as residence and wound type were factors associated with surgical site infections in the multivariable logistic regression analysis at a *p*-value of 0.05. For patients who had clean-contaminated and dirty wounds, the odds of surgical site infections were 12.81 times higher compared to those who had clean wounds (AOR = 12.81, 95%CI: 4.42 37.08). For rural dwellers, the odds of surgical site infections was decreased by 87% compared to urban residents (AOR = 0.13, 95%CI: 0.034 0.55) (Table 5).

**Table 5:** Binary logistic regression analysis to identify factors associated with surgical site infection among patients who undergone surgical procedure in private hospitals of Bahir Dar city, Ethiopia, May, 2018(*n* = 321)

Characteristics	SSI		COR(95%CI)	AOR(95% CI)
	Yes	No		
Residence				
Urban	18	207	1	1
Rural	3	93	0.37 (0.10 1.29)	0.13 (0.034 0.55)*
Sex				
Male	13	134	1	1
Female	8	166	0.49 (0.20 1.23)	0.40 (0.14 1.12)
Wound type				
Clean	7	235	1	1
Clean contaminated and dirty	14	65	7.23 (2.80 18.65)	12.81 (4.42 37.08)*
Length of hospital stay				
< 6 days	4	119	1	1
≥ 6 days	17	181	2.79 (0.91 8.50)	2.95 (0.85 10.19)
Duration of surgical procedure				
≤ 30 min	4	136	1	1
31–60 min	11	122	3.06 (0.95 9.87)	2.08 (0.58 7.44)
> 60 min	6	42	4.85 (1.30 18.03)	3.37 (0.80 14.20)

*Shows *P*-value less than 0.05

## Discussion

This study revealed that the overall prevalence of surgical site infections in the hospitals was 9.9% with (95%CI: 7.8 12.4). This finding was consistent with those of studies conducted at Suhil (11.1%) [13] and Asela (9.4%) [14] and Feleghiwot hospitals of Ethiopia (9.4%) [15] and at public hospitals in Tanzania (12%) [8]. However, this finding was lower than that of a study conducted at Hawassa hospital, Ethiopia (19.1%) [16], Gahanna (39%) [17]), Nigeria 38.1% [18] and India 21.66% [19]. The possible explanations might be differences in health care systems and socio-demographic characteristics of patients. The other difference might be the study population; in Nigeria and India surgical site infection assessments were done on patients who underwent laparotomy (abdominal surgery) which might have led to the overestimation of the prevalence. Nearly one-third of our procedures were Cesarean sections which had a lower risk for surgical site infections.

This study also revealed that the magnitude of surgical site infections varied by being 13.4% in the public and 6.5% in private hospitals. The possible explanations might be that most (98.4%) of the surgical operations in private hospitals were performed by senior surgeons, while only28.97% of the patients in the public hospital were operated on by senior surgeons. Moreover, private hospitals are relatively clean and implement strict infection prevention strategies to increase patient satisfaction. In contrast, public hospitals serve the population in sub-standard conditions with poor infection prevention practices due to high patient load.

In general, income level, wound type, length of hospital stay≥6 days, longer operation procedure duration and procedures done by residents and GPs were predictors of the overall surgical site infections. On another hand, clean-contaminated wound type, length of hospital stay, duration of operation procedures were factors with associated surgical site infections in procedures in public hospitals, whereas residence and wound type were predictors of SSI in private hospitals.

Average monthly income of between ETB 1601–2500 and more than 2500 was associated with an increased occurrence of surgical site infections. This might be so because individuals with high income are often associated with co-morbidities like DM which alter immunity and increase susceptibility to nosocomial infections. In our study, most patients (82.55%) with chronic co-morbidities had income greater than ETB 1601.

Thus, for patients who came from rural areas and had surgical procedures in private hospitals, the odds of surgical site infections decreased compared to urban residents. This might be so because in the prevalence of co-comorbidities like Diabetes and HIV is lower in rural areas. Nearly two-thirds of the elective procedures done were on rural residents which decreased the occurrence of surgical site infections. In addition, practices such as bathing before procedures which decolonize microbes on the body might contribute to reduced occurrences of SSI [20].

Clean-contaminated and dirty wounds were more likely to be associated with surgical site infections in private, public, and overall hospitals. This finding is supported by findings at Hawassa and Suhil hospitals of Ethiopia [13], St. Petersburg, Russian Federation [21], Cameroon [22] and Tanzania [23]. This might be due to the fact that dirty wounds have more probability of developing infections than clean wounds in similar care practices if there is contamination that might lead to infections.

A prolonged operation time that exceeds 1 h was associated with increased surgical site infections in both groups of hospitals This finding was consistent with previous studies [22, 24]. This might be due to the fact that prolonged operations lead to more exposure to environments which might increase the probability of contamination. In addition, prolonged operation time may increase leakage from adjacent organs.

Generally, prolonged hospital stay was associated with increased surgical site infections among patients who underwent surgical procedures. This finding was consistent and was supported by another study [12, 17, 19, 25, 26]. Longer hospital stay increased the risk of healthcare-associated nosocomial infections. This study also revealed that longer hospital stays at public hospitals were also associated with increased occurrences of surgical site infections. This might be because the public hospital was visited by a large number of patients accompanied by poor infection prevention practices. This led to increased occurrences of surgical site infections.

Surgical procedures performed by residents and general practitioners were associated with increased surgical site infections compared to procedures done by surgeons. This finding was concordant with results in Cameron [22, 27]. This could be due to the fact that surgical outcome depends on professional skills and experiences. Residents and general practitioners’ limited experience and skills might have contributed to increased occurrences of complications, including surgical site infections.

This study shares the limitations of retrospective study designs that are secondary data sources which have considerable missing. Surgical site infection diagnosis was merely made by clinical manifestations which might have introduced bias that led to under or overestimations of conditions. In addition, microbiological isolations were not done for all surgical site infection, and that might have introduced bias in the ascertainment of outcomes.

## Conclusion

The prevalence of surgical site infections was high, and a significant difference was observed between public and private hospitals. High average monthly income, clean-contaminated and dirty wounds, prolonged operations, hospital stays, and procedures are done by residents and general practitioners were associated with surgical site infections. Specifically, clean-contaminated and dirty wounds, prolonged operations and hospital stays were predictors of surgical site infections in public hospitals, whereas rural dwelling and clean-contaminated and dirty wound also predicted surgical site infections in private hospitals.

## Data Availability

The datasets used during the current study are available from the corresponding author on reasonable request.

## Abbreviations

AOR: Adjusted odds ratio
ASA: American society of anesthesiologist score
DM: Diabetes mellitus
ENT: Eye nose and throat
ETB: Ethiopia birr
HAI: Health care-associated infections
HIV: Human immune virus
ICU: Intensive care unit
OPD: Outpatient department
OR: Operation room
SPSS: Statistical package for social science
SSIs: Surgical site infections
Study US: The United States
WHO: World Health Organization
